# Editorial: Methods in industrial biotechnology and bioprocess engineering—Microalgae as a source of valuable compounds

**DOI:** 10.3389/fbioe.2023.1141280

**Published:** 2023-02-01

**Authors:** Ramachandran Sivaramakrishnan, Govindarajan Ramadoss, Imran Y. Pancha, Aran Incharoensakdi

**Affiliations:** ^1^ Laboratory of Cyanobacterial Biotechnology, Department of Biochemistry, Faculty of Science, Chulalongkorn University, Bangkok, Thailand; ^2^ School of Chemical and Biotechnology, SASTRA University, Thanjavur, Tamil Nadu, India; ^3^ Department of Industrial Biotechnology, Gujarat Biotechnology University, Gandhinagar, Gujarat, India; ^4^ Academy of Science, Royal Society of Thailand, Bangkok, Thailand

**Keywords:** biofuels, cyanobacteria, inhibitors, microalgae, wastewater

Industrial biotechnology deals with the technology employing living cells such as bacteria, yeast, fungi, algae and the important cellular products like enzymes responsible for the synthesis of other valuable components with respect to the industrial importance. To address the serious issues in the modern industry, biotechnological processes are among the most favorable methods. Bioprocess engineering in biotechnology helps in manufacturing different biological products and in enhancing the quality of the end product. The developments in the field of bioprocess engineering and industrial biotechnology help in deciphering the production of various products and parts of cells from the microbes. These products include the industrially important compounds such as drugs, chemicals, fuels, fertilizers, and for the pharmaceutical, chemical, cosmetic and food industries. In addition, recent advancements in industrial biotechnology also address the greenhouse gas emissions by utilizing and converting renewable raw materials which can produce the alternative fuels. This Research Topic gathers microalgae-based studies, which highlight the novel methods to enhance the biohydrogen production in dark fermentative mode, as well as the isolation of a novel cyanobacterium with biotechnological potential. In addition, one review article discusses the wastewater treatment by microalgae with regard to economic and environmental aspects.

The first article of this Research Topic Pansook et al. involved the production of hydrogen by *Aphanothece halophytica*. Authors used different inhibitors to direct electron flow in the metabolism to their choice. Metabolic inhibitors have capabilities to direct the electron flow towards photosystem II that can allow for the increase in hydrogen production. Different inhibitors such as (2-chloro4-ethylamino-6-isopropylamino-1,3,5-triazine (atrazine), N-(phosphonomethyl)-glycine (glyphosate), 3-(3,4-dichlorophenyl)-1,1-dimethylurea (DCMU), 2-chloro-4,6-diethylamino1,3,5-triazine (simazine), malonic acid, sodium azide, rotenone, 2,4-dinitrophenol, sodium arsenate and glyceraldehyde were used to enhance the hydrogen production. The inhibitor simazine showed high potential towards cyanobacterial hydrogen production with nearly 40-fold increase in the hydrogen production. However, high simazine concentration decreased the biomass and chlorophyll content. Hence, appropriate simazine concentration should be used to maintain biomass content for the higher hydrogen production. Treatment with simazine led to a decrease of oxygen production by the cells resulting in an increase of hydrogen production. Nevertheless, it is noted that prolonged incubation of cells with simazine showed toxicity towards the cells and decreased the biomass and chlorophyll content.

With an alternative approach, the hydrogen production was enhanced by the nutrient limitation Chinchusak et al. Nutrients such as phosphorus and sulfur limtiation did not show any changes in the hydrogen production yield and hydrogenase activity. However, key nutrients like potassium and nitrogen limitation enhanced the hydrogen production yield and hydrogenase activity of *A. halophytica*. Authors studied the oxygen evolution level and found that the nitrogen and potassium limitation decreased the oxygen evolution level. On the other hand, nitrogen and potassium limitation enhanced the oxygen respiration which controls the oxygen content in the medium and contributes to the hydrogen production yield. The glycogen content was also evaluated by the authors showing the nitrogen and potassium limitation increased the glycogen content. In addition, the nutrient limitaiton also enhance the pyruvate kinase activity which contributes to the hydrogen production yield. Finally, authors evaluated the transcriptome analysis of genes engaged in cell metabolism towards hydrogen production. Most of the genes participating in the carbon and nitrogen assimilation, and photosynthetic and oxidative pentose pathways were upregulated under the nitrogen and potassium limitation conditions, whereas the genes engaged in the Calvin-Basham-Benson cycle and glycolysis pathway were downregulated. After all, the authors suggested that the nitrogen and potassium limitation should be the favorable method to produce sustainable hydrogen from *A. halophytica*.

On the other aspect, Koch et al. isolated and characterized the filamentous phototactic cyanobacterium *Phormidium yuhuli* AB48 that showed the efficient antibiotic resistance. The growth characteristics such as carbon and nitrogen utilization, pH and salinity tolerance, and antibiotic resistance were enhanced by using proteomics and metabolomics. The isolated organism was sequenced and confirmed as *P. yuhuli* AB48 using phylogenetic analysis. The authors studied the morphology and growth characteristics of the isolated cyanobacterium. They found that 1 mM NaCl and 10 mM NaOH treatment showed high biomass yield. The addition of NaCl and NaOH generated oxidative stress to the cells and altered the cell structures. In addition, the cells increased the exopolysaccharides production to protect against the oxidative stress generated by NaCl and NaOH. The authors found that the isolated *P. yuhuli* AB48 showed strong resistance towards various antibiotics. The results confirmed that the isolated strain did not produce the cyanobacterial toxins and it could be used as the food source or fertilizer component. The multi-omics analysis confirmed that the isolated strain can survive under extreme conditions.

Regarding microalgae-based wastewater treatment with respect to economic and environmental sustainability, Srimongkol et al. reviewed the current scenario and prospects. The authors discussed wastewater treatment using microalgae which produces valuable biomolecules such as lipids, carbohydrates, proteins and pigments. Microalgae grown in wastewater can be a rich source of lipids for biodiesel or omega-3 fatty acids, carbohydrates for bioethanol or biopolymer, proteins for animal feed or health supplements, and pigments for cosmetics and natural dyes production. Authors further discussed the integrated microalgae biorefinery for the wastewater treatment and high-value compound production. The valuable biofuels production such as biodiesel, bioethanol, biohydrogen and biogas from the microalgae produced from the wastewater treatment was presented. The spent biomass could be further utilized to produce other valuable compounds such as antiviral agents, having anti-inflammatory, anti-cancer, and antioxidant activities. The nutrient rich wastewater for the microalgae cultivation can contribute to the circular economy and environment sustainability process. In addition, authors reviewed the coupling of carbon dioxide mitigation and wastewater treatment using microalgae. The discussion on challenges involved in the wastewater treatment in algal biorefinery opens the new path towards commercialization. It is necessary to commercialize the microalgae biorefinery on a large scale by using wastewater treatment plants for the circular economy and environmental sustainability.

In summary, the four articles of this Research Topic will be useful for researchers working on microalgae as a reference for their own innovative ideas in the field of industrial biotechnology.

